# Genome characteristics and type IV effector protein repertoire of *Coxiella burnetii* depend rather on Genomic Groups than on host species

**DOI:** 10.1186/s12866-026-04897-w

**Published:** 2026-04-22

**Authors:** Hanka Brangsch, Christian Berens, Selina Fuchs, Stephen Fitzgerald, Tom N. McNeilly, Anja Lührmann, Katja Mertens-Scholz

**Affiliations:** 1 Friedrich-Loeffler-Institut, Institut für bakterielle Infektionen und Zoonosen, Jena, Germany; 2 Friedrich-Loeffler-Institut, Institut für molekulare Pathogenese, Jena, Germany; 3 Institute for Infectious Diseases and Infection Control and Center for Sepsis Care and Control (CSCC), Jena University Hospital, Jena, Germany; 4 Moredun Research Institute, Penicuik, UK; 5 Mikrobiologisches Institut, Universitätsklinikum Erlangen, Friedrich-Alexander-Universität Erlangen-Nürnberg, Erlangen, Germany

**Keywords:** *Coxiella burnetii*, genotyping, T4BSS effector proteins, pangenome, SNP, host preference, Genomic Groups, type IV secretion system

## Abstract

**Background:**

Q fever is a zoonotic disease with virtually worldwide dissemination. Its bacterial agent, *Coxiella burnetii*, is primarily found in cattle and small ruminants. Disease manifestation is highly variable, i.e. asymptomatic, acute or chronic in humans, and subclinical or present as reproductive disorders in ruminants. Different genomic lineages of *C. burnetii* have been recognized and are considered to show host preferences and influence the disease outcome. The virulence of *C. burnetii* is essentially determined by effector proteins that modulate host cell processes, allowing the bacterium to persist and proliferate in the host. Thus, these effectors have been suggested to play a role in the presumed host specificity and disease manifestation.

**Results:**

In the present study, a comprehensive set of 140 *C. burnetii* genomes from ten Genomic Groups (GGs) and various hosts was studied bioinformatically to determine if there was an association between their genomic characteristics, including the effector protein repertoire, and their isolation source. The differences in genome size, IS1111 count, number of coding sequences, accessory genome and others could be attributed to lineage-specific traits. Likewise, the GGs showed conserved sets of effector proteins, although intra-lineage variances were high in GGIV. Several effector proteins, e.g. Cem8 (CBU_1634a) and CBU_0469, were highly conserved, while CBU_2007 showed a remarkably high number of sequence variants.

**Conclusions:**

*C. burnetii* exhibits genomic diversity that aligns with phylotypes rather than host species, suggesting that genomic traits as well as host factors influence disease outcome rather than a host species specific adaptation.

**Supplementary Information:**

The online version contains supplementary material available at 10.1186/s12866-026-04897-w.

## Background

*Coxiella burnetii* is a Gram-negative, obligate intracellular zoonotic pathogen and the etiological agent of Q (query) fever in humans or coxiellosis in animals. Q fever is distributed worldwide, except in New Zealand, and has been categorized as a priority zoonotic disease by the European Food Safety Authority (EFSA) since 2023. 

*C. burnetii* displays a broad host spectrum and infects a variety of species, including humans, domestic and wild animals, ticks and birds [1]. Disease manifestation differs between humans and animals. In humans, an infection remains often asymptomatic. About 40–50% of infected individuals develop a mild flu-like illness. However, in some patients, the infection progresses to an atypical pneumonia or hepatitis. A small percentage (2–5%) of infected individuals develop chronic Q fever, months or years after the initial infection. Chronic Q fever is mainly characterized by a potentially fatal endocarditis [1]. Ruminants, such as cattle, sheep or goats, are considered the main reservoir and source of human *C. burnetii* infections. Infections in sheep and goats are mostly asymptomatic. However, weak offspring and late term abortions do occur, with the abortion rate being higher in goats than in sheep. Fertility problems are common in cattle, but the symptoms are more varied [2]. Infected animals shed the pathogen through their feces and milk, but primarily through birthing products [1]. Humans are mainly infected by inhalation of contaminated dust, with less than ten bacteria being sufficient to cause disease [3].

Differences in the disease manifestations observed led to the assumption of an isolate-specific virulence and to the establishment of six Genomic Groups (GGI-VI) by restriction fragment length polymorphism (RFLP) analysis in the early 1990s [4]. These genomic groups (GGs) correlated with disease manifestations: isolates of GGI to GGIII originated mainly from patients with acute Q fever whereas GGIV and GGV isolates were associated with chronic human Q fever cases.

This original genomic grouping is still valid and was extended by modern typing methods, such as multispacer sequence typing (MST) and core genome single nucleotide polymorphism (SNP) typing [5, 6]. In MST, the allelic states of ten genomic loci are determined, while SNP analysis investigates base differences between strains in the entire DNA sequence, allowing a detailed differentiation. The initial panel of six GGs was extended by subdivision of GGII (a-d) and GGIV (a-b) [5, 7], based on specific SNPs, and addition of GGVII and GGVIII [8]. Rodent infection models supported the hypothesis of a genomic profile-specific pathotype with GGI to GGIII isolates causing more severe clinical signs, whereas GGIV and GGV isolates caused no or only mild disease [9, 10]. Additionally, the increasing availability of genome sequencing data of *C. burnetii* isolates from various host species revealed that GGs are dominated further by isolates from certain hosts, e.g. GGIII is dominated by isolates from cattle, whereas goat isolates are more frequently found in GGII-b and human isolates in GGII-a, GGIV and GGV [5].

The genomes of *C. burnetii* isolates comprise a chromosome with ~ 2 million base pairs and a mean GC content of 42.6% [11, 12]. In total, the genome contains an estimated 2,134 coding elements, the exact number of which varies between different *C. burnetii* isolates [11]. In a recent study, 75 isolates were analyzed and grouped into 22 MST genotypes and 13 clusters [12, 13]. Importantly, all isolates analyzed contained genes encoding a type IVB secretion system (T4BSS) [12]. T4BSS are complex nanomachines that span the entire bacterial cell envelope and deliver DNA or effector proteins into the host cell environment [14, 15]. Effector proteins manipulate a variety of host cell pathways to ensure bacterial propagation. Thus, the T4BSS is integral for bacterial virulence [16]. *C. burnetii* encodes 23 homologs of the 26 *Legionella pneumophila dot*/*icm* genes, that encode the T4BSS [17, 18]. The T4BSSs of these two pathogens are not only structurally, but also functionally similar. *C. burnetii* lacking a functional T4BSS is unable to replicate intracellularly [19, 20], demonstrating the importance of this secretion system for virulence. To date, ~ 150 *C. burnetii* T4BSS effector proteins have been identified, but only few have assigned functions [21, 22]. These effectors promote biogenesis of the *C. burnetii*-containing vacuole (CCV), interfere with vesicular trafficking, maintain host cell survival and manipulate host immune defenses [22, 23]. The CCV is established after uptake of *C. burnetii* into the host cell. The nascent CCV has a neutral pH and is decorated with early endosomal marker proteins. Maturing CCVs are phagolysosome-like compartments with an acidic pH of ~ 4–5 [24–26]. These acidic conditions induce the translocation of T4BSS effector proteins into the host cell [27], which in turn allows completion of CCV maturation into a large, replication-competent vacuole, and modulation of the host cell in favor of the pathogen.

Several studies have demonstrated considerable heterogeneity among the *C. burnetii* effector protein profiles from different isolates [18, 28–31]. In a study, in which the repertoire of effector proteins was compared in five isolates, only 44 out of the 143 effector proteins analyzed were present and intact in all five strains [18].

Here, we analyzed the genomes of 140 *C. burnetii* isolates to determine whether affiliation to a GG and/or the T4BSS effector protein repertoire as well as their secretion system might allow the prediction of virulence potential or host species specificity of an isolate. The dataset comprised 102 publicly available genomes and 38 recently sequenced *C. burnetii* isolates from Germany. All GGs were represented, except for GGII-d, GGVII and GGVIII, as well as common host species, such as cattle, goats, sheep, humans, ticks, and rodents. They originated from acute and chronic Q fever cases or from afterbirth material and milk from ruminants.

## Results

### Selection of *C. burnetii* genome sequences

To generate a comprehensive and high-quality dataset of *C. burnetii* genomes, genomic data from the NCBI Short Read Archive (SRA) (*n* = 110) and the RefSeq database (*n* = 150) was retrieved. Further, 38 isolates from the *C. burnetii* strain collection of the Friedrich-Loeffler-Institut were included that had been collected in Germany and the Netherlands, from small ruminants, cattle and, in one instance, from a patient, between 1989 and 2021 (Additional file 1 - Table S1). These served to complement the publicly available genomic data. These isolates were sequenced by Illumina and Nanopore technologies.

All *C. burnetii* datasets were assessed for their quality and completeness. Metagenomic datasets were removed because of low *C. burnetii*-specific read counts. Duplicates of identical strains were also excluded. Overall, 127 public data sets (SRA *n* = 31; RefSeq *n* = 96) of high quality representing individual isolates were chosen for further analyses (Additional file 1 - Table S1). Almost half of these (*n* = 62) originated from humans.

All assemblies or the corresponding read data were subjected to SNP typing together with the SRA data for excluding duplicate strains. The core genome SNP alignment contained 14,589 SNPs and 0 to 5,681 nucleotide differences were observed between individual strains. Duplicates of identical strains were removed (*n* = 25), leaving 140 unique strains in the final dataset.

For all downstream analyses, 102 unique public data and 38 new genome sequence data (*n* = 140) sets were used. Many of these strains were of human origin (*n* = 58), but strains originating from cattle (*n* = 33), goats (*n* = 22) and sheep (*n* = 12) were also included. Furthermore, one strain each had been isolated from a dog, a mouse, the soil and a not-specified ruminant, respectively. Eight strains came from ticks and three from kangaroo rats. The majority of the strains (*n* = 99) had been isolated in Europe, particularly in France and Germany.

### Selection of *C. burnetii* effectors

To assess if the repertoire of effector genes varied among isolates, and if any observed differences correlated with potential adaptation to specific hosts and/or disease manifestation, a comprehensive literature survey was conducted to gather data on known *C. burnetii* effector proteins. Overall, a total of 156 effector genes comprising 146 chromosomally encoded and ten plasmid encoded genes were identified in the reference strain Nine Mile I (Additional file 2 - Table S2). Of these, two could not be found in the NMI reference strain (CBU_0088, CBU_1251) and 23 have been marked as discontinued in NCBI. Additionally, homologues of effector-coding genes of strain Nine Mile I were searched in the reference strains Dugway 5J108-111, CbuG_Q212, CbuK_Q154 and RSA331, resulting in the identification of 438 homologous effector sequences across all strains (Additional file 2 - Table S2).

### Placement of the strains in the *C. burnetii* phylogeny

**Fig. 1:**
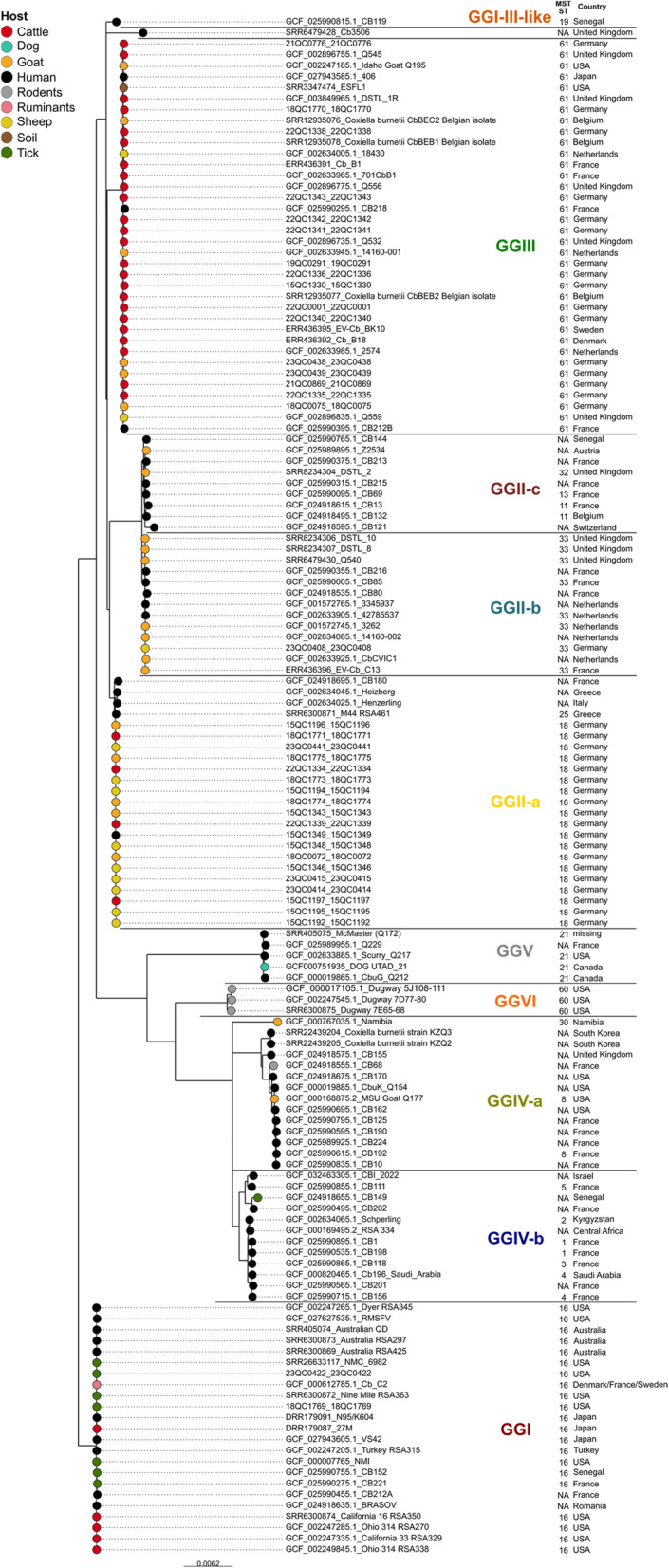
Maximum likelihood tree based on cgSNP alignment of 140 *C. burnetii* strains with their sequence accessions and their strain designations for public data. Leaf colors indicate the host of isolation (circles). GGs are separated by horizontal lines. In silico MST sequence type and the country of origin are shown at the branch tips. The scale bar indicates base substitutions per alignment site

The strain selection should represent a wide range of known *C. burnetii* phylotypes for gaining a comprehensive insight in effector protein variation. Thus, the genomic diversity of the isolate or genome datasets was first assessed by in silico MST analysis followed by linking to GGs according to Hemsley et al. [6]. In total, 18 different sequence types (STs) were identified, mostly ST61 (*n* = 38), ST16 (*n* = 28) and ST18 (*n* = 19). However, various novel alleles were found, so that a ST could not be assigned to 34 strains (Fig. 1, Additional file 1 - Table S1). Based on the MST results, the strains were also assigned to a GG, showing that our dataset included strains from ten of the 13 known GGs.

The subsequent core genome SNP (cgSNP) analysis confirmed the MST and GG results, as all strains with an identical MST ST and the same GG clustered together (Fig. 1). The size of the core genome SNP alignment totaled 15,343 nucleotides; more than in the previous alignment for quality control, accounting for the higher quality of the final dataset.

Cattle isolates dominated GGIII, whereas sheep- and goat-associated strains were primarily found in GGII-a and GGII-b. Remarkably, almost no animal isolates were found in GGIV and GGV, i.e. these groups were dominated by human isolates. However, most GGs (GGI, GGII-a/b, GGIII, GGIV-a/b) were composed of strains that had been isolated from three to four different host species, while two GGs (GGII-c, GGV) were detected in only two host species each (human and goat or human and dog, respectively) and GGVI exclusively comprised isolates from a single host species (kangaroo rat). The human isolate Cb3506 from the United Kingdom, which was located on the same branch as GGII and GGIII, could neither be assigned to an MST ST, nor placed within a GG.

Collectively, this dataset represented the majority of known *C. burnetii* GGs. Furthermore, GGs previously determined to be dominated by specific host species were confirmed, although most GGs were associated with three different host species.

### Genome characterization and pangenome analysis

#### Genome characterization

Using the cgSNP typing approach, almost all strains were assigned to a GG. Thus, in the following analyses, the genomes of the groups could be characterized collectively and differences between these groups were assessed.

The genome sizes ranged from 1,955,281 bp in one human isolate, that could not be assigned to a GG, to 2,212,937 bp in the GGVI reference strain Dugway 5J108-111. In GGV, the mean genome size was lowest (appr. 1,992 kbp) (Table 1). The variation in genome size was highest in GGII-b, GGII-c and GGIV-a. No connection between genome size and the associated host was apparent (Additional file 3 - Figure S1). The number of coding sequences detected ranged from 1871 in a strain from GGIV-b to 2248 in a GGII-b strain. The lowest mean number of coding sequences (CDSs) was found in GGIV-b, while the genomes of GGII-a showed not only the highest number of CDSs, but also of pseudogenes, i.e. non-protein-coding genes. GGI genomes had the least number of pseudogenes (*n* = 46 ± 2), even less than the Dugway strains from GGVI (*n* = 58 ± 8) (Additional file 1 - Table S1). The number of genes without significant similarities to genes with known function or known domain (“hypotheticals”) was lowest in GGVI and highest in the GGII subgroups.

**Table 1:** Genome characteristics of the 140 *C. burnetii* strains analyzed, according to their affiliation with a Genomic Group (GG). Given are the arithmetic mean and its standard deviation (SD)

GG	#*	Genome size		CDSs		Pseudogenes		Hypotheticals	
		Mean	SD	Mean	SD	Mean	SD	Mean	SD
I	23	2,012,857	18,387	2018	30	46	2	305	11
II-a	23	2,044,679	23,953	2100	51	111	15	353	23
II-b	13	2,032,638	44,789	2073	92	102	22	359	46
II-c	9	2,041,355	44,428	2084	115	103	27	361	57
III	36	2,017,346	15,564	2044	26	80	9	330	10
I-III-like	1	2,018,549	-	2024	-	59	-	303	-
IV-a	13	2,044,119	43,717	2013	80	108	17	318	34
IV-b	13	2,043,400	26,878	1980	43	83	8	301	32
V	5	1,992,946	15,407	2009	28	99	7	346	13
VI	3	2,198,908	9,920	2079	20	58	8	272	3
NA	1	1,955,281	-	1989	-	81	-	325	-

*# number of isolates or genome data sets included

These data showed that genome size and the number of CDS or pseudogenes differ between the GGs. No correlation between the number of pseudogenes and genome size was found, i.e. the number of pseudogenes was highest in GGs (GGII and IV-a) with the most variation in genome size, but isolates with small genomes (GGV) harbored a similarly large number of pseudogenes.

The genomic GC content was lowest in GGVI (mean: 42.35%), whereas for most GGs (GGI, GGIII, GGIV, GGV) the mean GC content was 42.55% to 42.56%. The subgroups of GGII showed slightly higher GC contents (mean: 42.62% to 42.68%).

#### Insertion elements

**Fig. 2:**
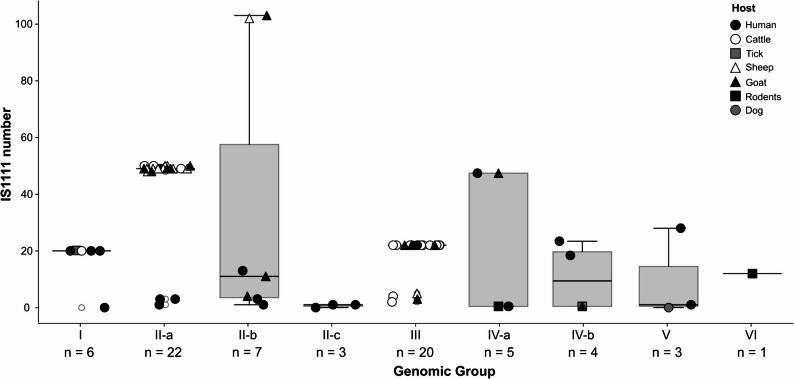
Box plot showing the distribution of IS1111 elements detected in 71 *C. burnetii* genome assemblies that were almost closed (max. three contigs). Shapes and filling of the data points indicate the host of the strain. The sample numbers are indicated below the labels on the x-axis

Insertion elements (IS), especially the IS1111 element, are associated with genome rearrangements and genome plasticity in *C. burnetii*. Insertion events can introduce gene disruption, small indels or mutations and have been associated with a pathoadaptive evolutionary process [32]. Therefore, the number and type of IS elements were determined and compared among strains or genomic datasets of different GGs. Only genomes with a maximum of three contigs (*n* = 71) were analyzed, as fragmented assemblies often show breaks at repetitive elements and, thus, the number of IS elements could be overestimated. In these 71 genomes, four to 114 IS elements belonging to eight families (IS110, IS1634, IS3, IS30, ISAS1, ISNCY, IS4, IS481) were detected (Additional file 4 - Table S3). Dugway 5J108-111 was the only strain analyzed in which an IS4 sequence was detected. All strains had one copy of ISNCY. Elements of the IS481 family were only detected in the genomes of GGIV-a, GGV and GGVI, but not in GGIV-b, except for strain Namibia (MST30). IS1111, the only known representative of family IS110 in *C. burnetii*, was identified up to 103 times in one genome (goat isolate 3262 of GGII-b) (Additional file 4 - Table S3). However, to our surprise, ISEScan did not detect IS1111 elements in three genomes (strains DOG UTAD (GGV), CB121 (GGII-c), BRASOV (GGI)). Checking the annotation files revealed that the strains did harbor transposons, but apparently the degree of sequence identity at the nucleotide level was not high enough to be detected by the bioinformatic tool used. Additionally, only a single copy of IS1111 was found in ten strains. The distribution of the number of IS1111 elements within and between the GGs is displayed in Fig. 2. Most GGs were consistent in their IS1111 copy number, particularly GGI, GGII-a and GGIII, where most isolates harbored around 20, 50 and 22 IS1111 copies, respectively. Two isolates from goat and sheep, respectively, of GGII-b stood out, as 102 and 103 IS1111 copies were detected. A correlation between the number of IS elements and the host species was not apparent, e.g. isolates from goats were often among the strains with the highest or the lowest number of IS1111 copies.

Overall, the differences observed in the number of IS1111 elements were mostly in accordance with the phylogenetic grouping of the strains, but no connection to host species was observed.

#### Pangenome analysis

**Fig. 3:**
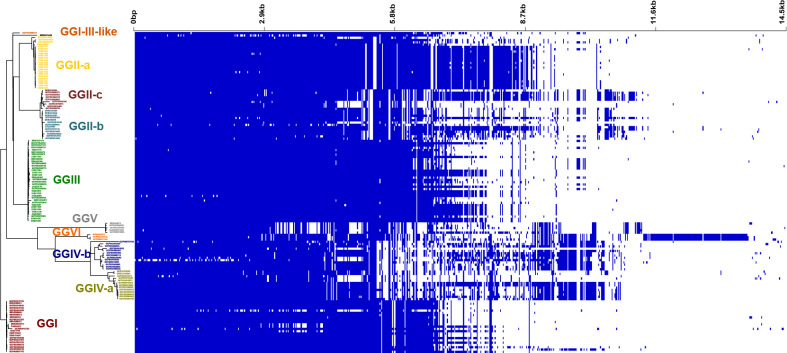
Pangenome analysis of 140 *C. burnetii* strains. The maximum likelihood tree was generated based on the core genes. The blue bars at the right indicate the presence of accessory genes. Isolates of the same GG are colored identically: red – GGI, yellow – GGII-a, turquoise – GGII-b, bordeaux – GGII-c, green – GGIII, olive – GGIV-a, blue – GGIV-b, grey – GGV, orange GGVI, ocher – GGI-III-like, black – no GG

Using the complete dataset of 140 genomes, a pangenome analysis was conducted (Table 2). The aim was to determine if a specific set of accessory genes could differentiate strains from different GGs and/or strains from identical hosts or if differences primarily occurred in genes conserved across the *C. burnetii* phylogeny. Of the 2237 total genes detected, 72.6% were found in at least 99% of the strains and constituted the core genome in this study. Less than 10% of the genes were only found in less than 20 genomes each (15%). In agreement with the previous cgSNP analysis, a phylogeny based on only core genes identified in the pangenome analysis generated the same GG clusters (Fig. 3). The corresponding visualization of gene presence, displayed as blue bars in Fig. 3, indicated that each GG possessed a specific set of accessory genes. Particularly, the Dugway isolates of GGVI featured a large set of genes absent in other isolates. This Group also had the largest number of lineage-specific core genes (*n* = 1979) (Additional file 5 - Table S4), while GGIV-b harbored the lowest number of lineage-specific core genes (*n* = 1664). However, the percentages of core genes relative to the overall number of genes detected differed between the groups, with the fewest conserved genes in GGII-b.

**Table 2:** Result of the pangenome analysis of 140 *C. burnetii* genomes

Fraction	Definition	No.	%
Core genes	(99% <= strains < = 100%)	1624	72.6
Soft core genes	(95% <= strains < 99%)	100	4.5
Shell genes	(15% <= strains < 95%)	303	13.5
Cloud genes	(0% <= strains < 15%)	210	9.4
Total genes	(0% <= strains < = 100%)	2237	

**Fig. 4:**
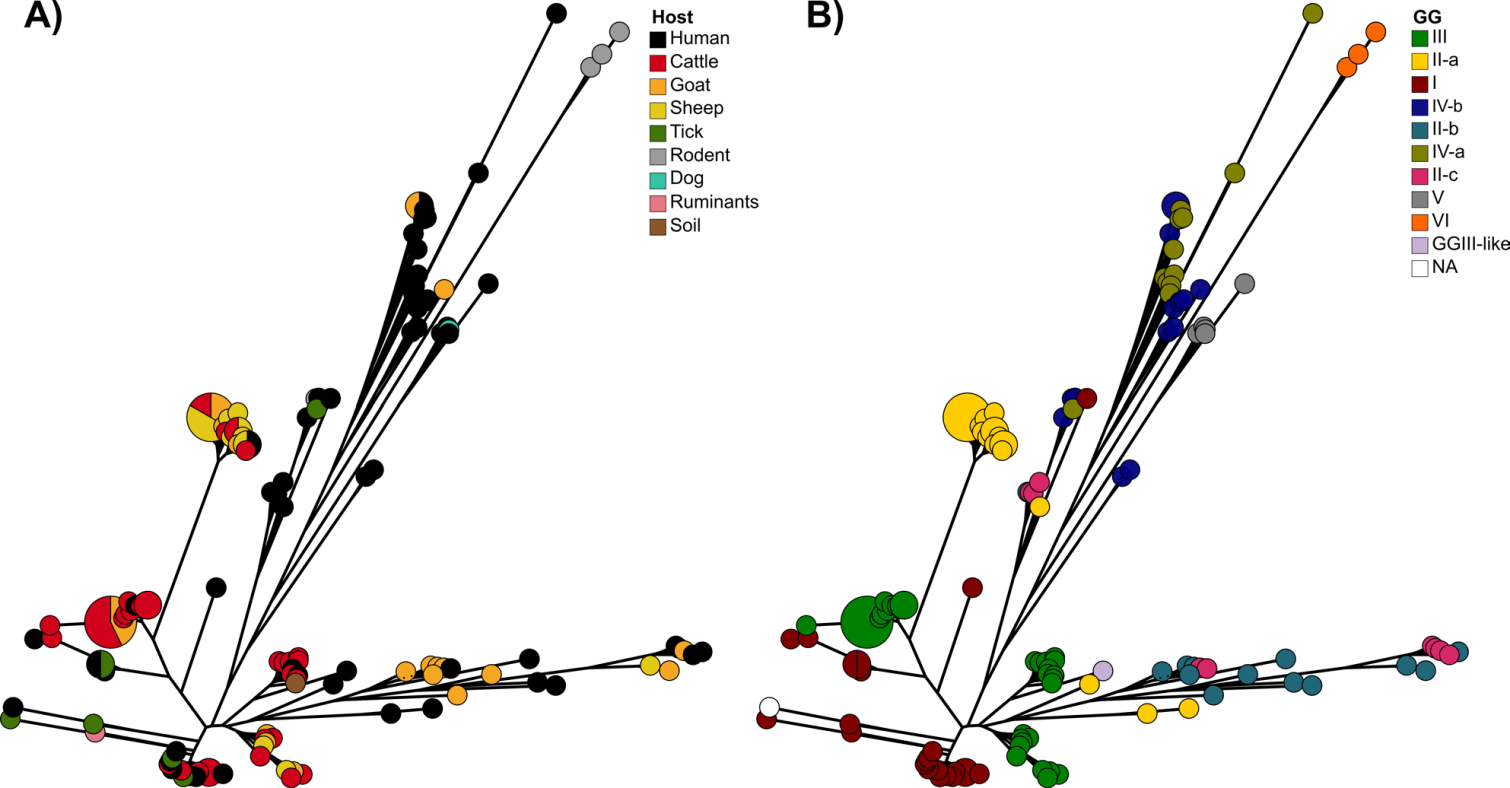
Neighbor joining trees based on the binary presence/absence information of 723 accessory genes in 140 *C. burnetii* strains. Colors correspond to the host (**A**) or the Genomic Group (GG) (**B**) of the strains

The presence/absence information of the accessory genes (*n* = 723), i.e. genes present in less than 140 genomes, was used for a Neighbor Joining analysis, to see if it correlated with GG or the source of isolation (Fig. 4). For this analysis, the gene sequences were not considered, only their presence (binary information). This analysis confirmed that the accessory genes found in the strains were determined by affiliation to a GG rather than to a host species, as clusters were formed by GG rather than by isolate origin. Remarkably, the accessory gene spectrum of the GGs was diverse and only a few coherent clusters were observed.

Taken together, the accessory genes among all strains analyzed clustered according to the GGs by cgSNP and pangenome analyses. Each GG possessed a specific set of accessory genes, but with a certain variability within the group.

### T4BSS effector protein variations

#### Nucleotide-level analysis of effectors

We next compared the effector gene and predicted protein sequences of all T4BSS effector proteins identified to assess if the effector gene repertoires of different *C. burnetii* isolates were associated with host species.

Across the 100 effector genes of the core genome, 1,213 variant positions were detected at the single nucleotide level. There was a high degree of similarity within the GGs, as all strains within the four largest groups (GGIII, GGII-a, GGII-b and GGI) differed by not more than seven bases within the effector gene regions. However, the SNP differences in GGIV-a and GGIV-b were higher (Table 3).

**Table 3:** Number of single nucleotide variants and predicted effector protein sequence variants unique to each Genomic Group at the core genome level (“genome”) and in gene positions corresponding to NMI effectors (“effector genes”). Numbers in brackets show results for GGIV-b when strain Namibia (MST ST30) is excluded from the group

Region	Total SNPs	I	II-a	II-b	II-c	III	I-III-like	IV-a	IV-b	V	VI
Genome	15,343	388	110	67	39	360	228	651	1 (151)	2614	1175
Effector genes	1,213	38	6	4	3	24	20	49	0 (13)	210	90
Effector proteins	-	25	12	3	2	16	19	12	3 (7)	60	67

**Fig. 5:**
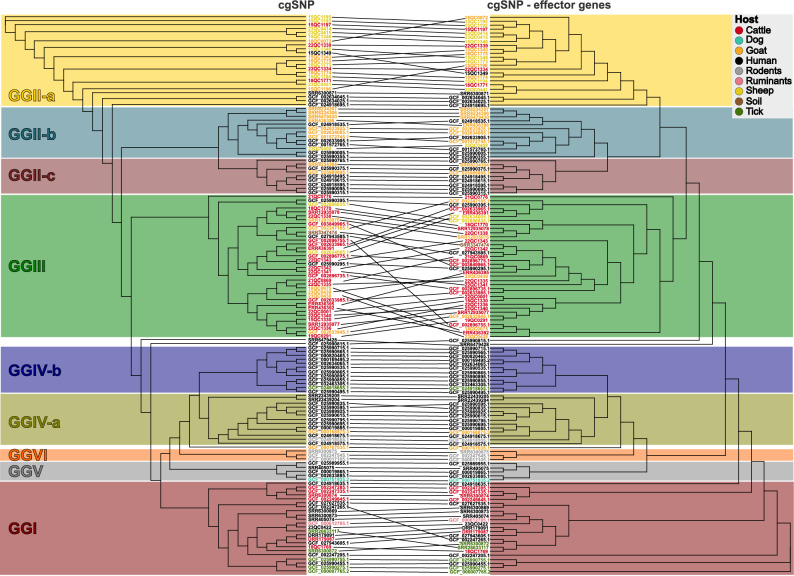
Tanglegram between the tree shown in Fig. 1 (left) and a maximum likelihood tree based on the cgSNPs detected in the effector protein-coding regions (right). The strain names are colored according to their host. Colored blocks indicate GGs: red – GGI, yellow – GGII-a, turquoise – GGII-b, bordeaux – GGII-c, green – GGIII, olive – GGIV-a, blue – GGIV-b, grey – GGV, orange GGVI, ocher – GGI-III-like, black – no GG

Direct comparison of core genome- and effector gene-based SNP typing (Fig. 5) revealed that the overall clustering of the GGs and the branch placement of most strains remained coherent. Only the GGIII strains and some GGI strains clustered differently within the group. This indicates a higher degree of variation regarding the SNP positions within this group.

The SNP positions in the core genome and in the effector-coding genes were screened for variants that were shared by all isolates of the same GG or from the same host, but differed in other strains (Additional file 6 - Table S5).

Unique SNPs were identified in the core genome region as well as in effector genes for all data sets with a GG assignment (Table 3). GGV had a high number of unique nucleotide variants and GGIV-b exhibited a single SNP (CBU_1459), but only when strain Namibia (MST ST30) was included. The latter did not cluster perfectly with other strains of GGIV-b. Only a few unique mutations were detected in the effector gene regions for GGII-a to -c.

No characteristic unique nucleotide variants were found when comparing the strains by their host species, not even when considering the complete core genome region. This finding was valid even when samples with potential accidental hosts or vectors were removed and only samples from cattle, human, rodent, sheep and goat were considered. Only for the rodent isolates, which all belonged to GGVI, unique SNPs were detected, which coincided with the previous results for GGVI.

Therefore, mutations in the core genome effector gene sequences coincided with the GG, allowing typing of isolates based on GG-unique SNPs. However, no association between the core genome effector gene sequences and host species was found.

#### Protein-level analysis of effector proteins

As differences in the nucleotide sequence do not necessarily translate to differences at the protein level, the effectors were also analyzed based on their predicted protein sequences to assess if sequence changes might impact protein functionality.

Seventeen of the 156 effector genes initially identified in the literature were annotated as pseudogenes in the RefSeq record, that do not have a translation product. Further, 25 effectors were not found due to discontinuation in the new RefSeq annotation version (v2) (Additional file 2 - Table S2). A comparison of the genome position of the remaining 131 coding sequences to the Bakta annotation of NMI showed, that all but one (CBU_0375) of the effectors were present and represented ORFs, even if they were pseudogenes in the original annotation. Additionally, the gene sequence of all NMI effectors from the RefSeq annotation were searched for in four reference strains representing different genomic groups: Dugway 5J108-111 (GGVI), CbuG_Q212 (V), CbuK_Q154 (IV-a) and RSA331 (II-a) (Additional file 7 - Figure S2). The protein sequences encoded by homologous genes were downloaded and a database was created. The gene products predicted from all strains in the dataset investigated were compared to this effector protein database and potential effector proteins were extracted.

**Fig. 6:**
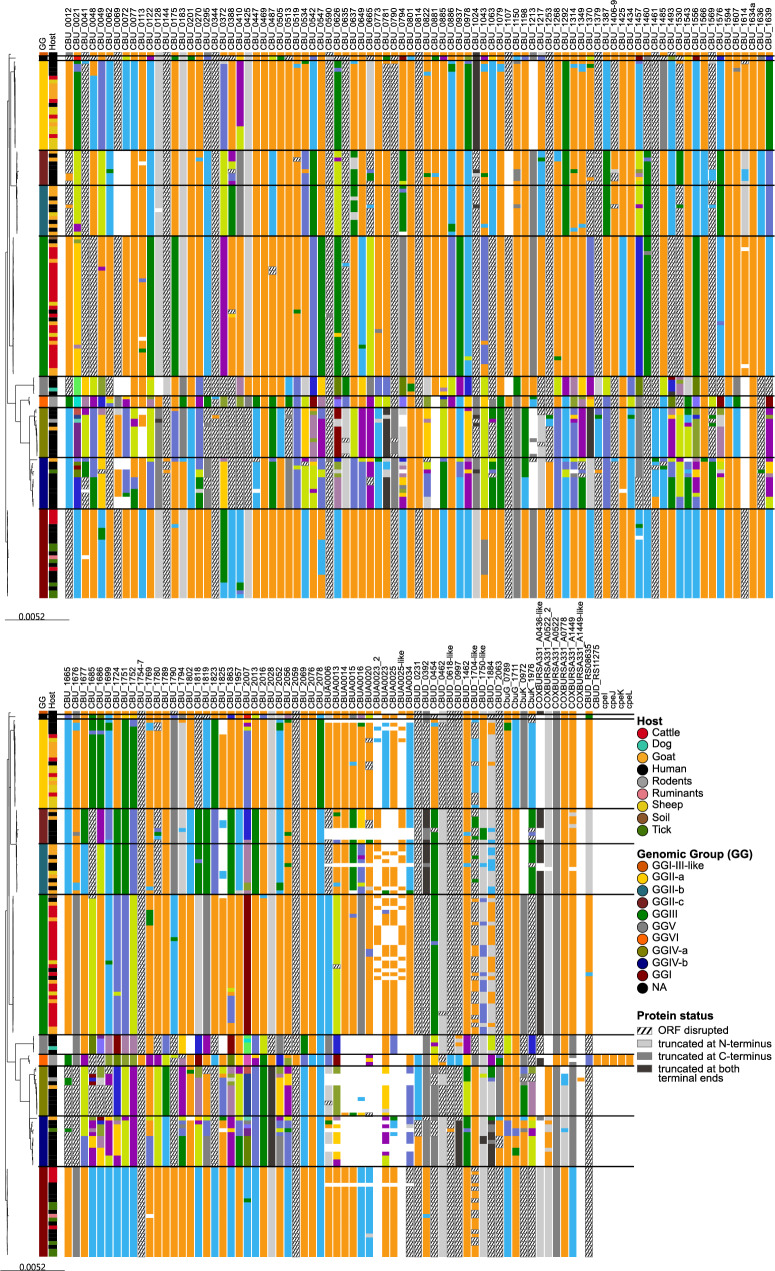
Presence and status of potential effector proteins in 140 *C. burnetii* strains. Block colors indicate differences in amino acid sequence, i.e. blocks with identical colors for one protein show sequence identity. Protein truncations are shaded grey and disruption is indicated by hatching. Host species and Genomic Group affiliation are given as the first two columns in the trees. The GGs are separated by black vertical lines

By this approach, 157 genomic loci and corresponding gene products were identified as potential effectors, which were investigated further. Each complete protein sequence variant was given a number to differentiate between them. This enumeration started anew for every effector. Incomplete sequences were labelled ‘truncated’. Figure 6 gives an overview over the variants observed for the effector proteins, sorted according to the pangenome phylogeny. If an effector protein was classified as truncated, it indicated that it was shorter than the longest observed sequence of this effector protein. Further, the coding region was classified as disrupted, if more than a single ORF was detected for this genomic locus in the pangenome analysis.

All 130 NMI effector proteins were found to be present in all strains, regardless of their truncation or disruption status (Additional file 8 - Table S6). When considering only full-length proteins, 35 NMI effectors were found in all strains and, additionally, all strains harbored three effectors of the strains Dugway (CBUD_1462) and CbuG (CbuG_0789, CbuG_1711).

In general, effector protein variants correlated with their respective GG, with some variation also occurring within a GG. To confirm this, a neighbor joining analysis was conducted with the effector sequence types (Additional file 9 - Figure S3). As expected, the strains clustered according to their GG. No connection to the host species was observed. Further, no unique sequence type was found when looking for host-specific effector types in the dataset. However, all GGs harbored effectors with unique sequence types (Table 3, Additional file 10 - Table S7). In GGII-b and II-c, only three and two effector variants were unique, respectively, which agreed with the frequent overlap of sequence types in GGII, and with the higher variance in these two sub-groups. Likewise, for GGIV-a and IV-b, a higher variance was observed, leading to low numbers of unique effector types. As observed for SNPs, excluding strain Namibia from GGIV-b increased the number of unique effector variants. In this case, strain Namibia showed 49 uniquely different effector sequences (Additional file 10 - Table S7).

Two effectors were conserved among all strains studied: CBU_0469 and CBU_1634a (Cem8). Also, CBU_1314a was highly conserved among all GGs, except for GGV, in which it was not detected, and for MST ST30 of GGIV-b, strain Namibia, that harbored a single amino acid exchange. Likewise, CBU_1594 (MceD) and CBU_2076 were identical in all strains, except for the GGVI genomes, in which they contained the C-terminal substitutions I109V and A97S, respectively.

Several effectors were not detected in all genomes. CBU_0072 (AnkA) was absent from GGII-b, GGII-c and GGVI as well as from a few GGIV-a and GGIV-b strains. The latter two groups and GGVI lacked CBU_0881 (CoxCC5), whereas CoxU1 (CBU_0814) was only intact in GGIV-a/b. Likewise, the genomes of GGVI possessed effectors that were missing or not intact in other strains: CBU_1107, CBU_1754-7 and CBU_2028.

Several effector proteins (*n* = 23), that were detected by screening with the effector protein database (Diamond database in Additional file 7 - Figure S2), could not be assigned to a NMI homologue, but showed high similarity to effector proteins from the strains Dugway 5J108-111, CbuG_Q212, CbuK_Q154 and RSA331. Thus, in Fig. 6, these proteins were named according to their respective match in the protein database. Interestingly, CBUD_0392, whose sequence was taken from the Dugway reference strain, was detected as truncated in GGVI and others, because a homologous protein was found in GGI, which was 159 aa longer at the C terminus than the Dugway reference protein. CBUD_0454, also from the Dugway reference, was disrupted in GGI, but intact in all GGII and GGIII strains, with one exception in GGII-c.

Six proteins (CBUD_RS11275, CBUD_RS08635, CpeI, CpeJ, CpeK, CpeL) were identified by their annotation but were not found by screening of the database or comparison to the NMI loci. CpeI to CpeL were annotated as Dot/Icm T4BSS effectors while CBUD_RS11275 and CBUD_RS08635 were described as ankyrin repeat domain-containing proteins. While CpeIL and CBUD_RS08635 were only detected in GGVI, variants of the 627 aa long CBUD_RS11275 were found in GGII, GGIII and GGVI. The protein was truncated at the N terminus in GGII-b and GGII-c.

When analyzing the effector repertoire at the protein level, several effectors were conserved in all GGs, a few effectors were present only in GGIV or absent in GGIV and GGV. Overall, a GG-specific effector sequence type pattern was observed, but a connection to the host species was not detected. However, this analysis did not assess functionality of the detected effector variants, which may impact virulence.

#### Detailed analysis of selected effector protein variants

Analyzing the genomic diversity of genes encoding putative effector proteins can help identify regions or amino acids essential for molecular activity [31]. Often, the C-terminal end (especially the last 20 amino acids) is essential for recognition and export by the T4BSS, while the N-terminal sequence may contribute to effector function or localization [30, 33, 34]. Thus, we analyzed the sequences of five potential T4BSS effector proteins, for which experimental data on their function and interaction with the host cell were available. These five effector proteins – CBU_0077 (MceA), CBU_0513 (CinF), CBU_0781 (AnkG), CBU_0822 (CbFic2) and CBU_2007 (Vice) – have been associated with interfering with apoptosis and host cell transcription, and/or are essential for intracellular replication and CCV biogenesis.

##### CBU_0077 (MceA)

**Fig. 7:**
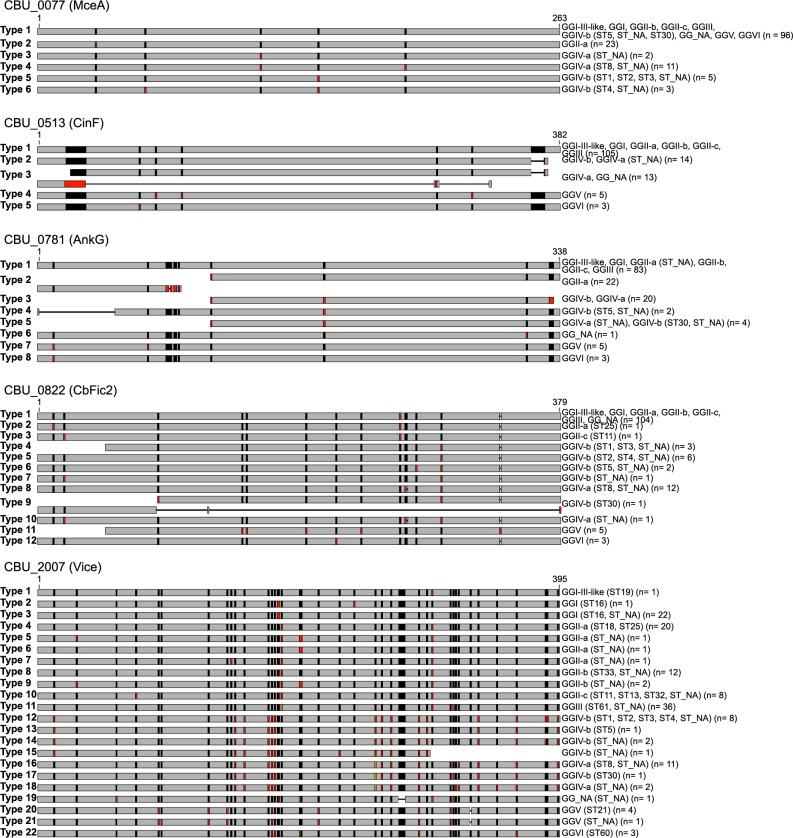
Schematic representation of protein sequence variants for five *C. burnetii* effectors, named according to the NMI annotation. Red, blue and yellow bars indicate amino acid changes relative to the consensus sequence (black bars). Dashes indicate deletions. At the right side, all GGs and/or MST sequence types with this protein variant are listed including the number of strains. Numbers above the first sequence bar of each protein indicate amino acid positions

MceA co-localizes with mitochondria but its function is unknown [35]. This effector was found in all genomes, with altogether six sequence variants (Fig. 7). While most GGs shared the protein sequence of NMI (GGI), GGII-a had a unique variant due to a substitution (A30S). The variability in GGIV-a and GGIV-b was higher, as both GGs showed two and three sequence variants, respectively. In GGIV-b, two variants (type 5 and type 6) had a K143E substitution and three strains an additional G55S exchange (type 6). In GGIV-a, CBU_0077 exhibited one substitution (Q113E) in two strains (type 3) and the majority of the strains (*n* = 11) also featured an S186N amino acid exchange (type 4). In all sequence variants, the C-terminus, which is likely required for secretion, was conserved. If the other single amino acid changes interfere with protein localization or function is unknown.

##### CBU_0513 (CinF)

Ectopically expressed CinF has cytoplasmic localization and is essential for intracellular replication [36]. Its sequence was intact and identical in GGI, GGII, GGIII, and GGI-III-like (Fig. 7). In GGIV, it was either intact or truncated at the C-terminal end by 19 aa residues. The later likely prevents T4BSS secretion, as the C-terminal ten amino acids contain the translocation signal [30]. In GGV, there were three substitutions (E87K, F106V, A318S) relative to the NMI reference, while the GGVI variant differed in only one position (W75R) from the majority of strains.

##### CBU_0781 (AnkG)

AnkG was one of the first *C. burnetii* T4BSS effector proteins identified [37]. Its task is the inhibition of host cell apoptosis and several amino acids within the N-terminal region were shown to be essential for its function [33, 34, 38]. In all strains of GGI, GGIII, GGII-b and GGII-c, the sequence of AnkG was intact and identical to the NMI reference protein (Fig. 7). However, eight sequence variants were found. In almost all strains of GGII-a, the ORF was disrupted, likely preventing function or secretion, except for strain CB180, in which the sequence was identical to the NMI reference protein. Full-length proteins of this effector showed one of two different amino acid mutations at the N-terminal end: amino acid position 11 encoded either isoleucine (variant of NMI reference) or leucine (GGV and GGVI), which could impact protein activity [34].

##### CBU_0822 (CbFic2)

Whether CbFic2 is a T4BSS effector protein has still to be determined, as experimental validation is lacking. Two domains were identified, an HTH domain (amino acids 304–362) required for nuclear localization and DNA binding, and a predicted Fic motif (amino acids 205–216), which are both essential for protein functionality [39]. CbFic2 was identical in most strains of GGI, GGII and GGIII, with two exceptions in GGII-a and GGII-c, respectively. These harboured each a substitution at the N-terminus: P12S or L20F. In GGVI, there were two substitutions relative to NMI: T217A and S263L. In GGIV, several different substitutions were observed. Further, in GGV, an insertion of serine at position 336 was observed. As this is located within the HTH domain, it might influence nuclear localization and/or DNA binding and thus protein activity. Importantly, none of the variants analysed was mutated in the amino acids 66 and/ or 205, which might alter the enzymatic activity of CbFic2 [39].

##### CBU_2007 (Vice)

Vice was identified as a cytoplasmic T4BSS effector protein [36, 40]. It was shown to be important for the establishment of a large CCV and for intracellular replication [22]. This effector exhibited the highest number of sequence variants, 22, among all detected effector proteins (Fig. 7). Ten of these were found in only a single strain each and three were only found in two strains each. Altogether, 38 variable amino acid positions were detected. Additionally, there was a deletion of a single amino acid in both sequence types of GGV (types 20 and 21) and a deletion in strain Cb3506 extending over five amino acids (type 19). In strain CBI_2022 of GGIV-a, the protein was truncated by 98 aa at the C-terminal end (type 15), likely preventing secretion [30]. The strains of Genomic Groups GGII-c, GGIII and GGIV were the only ones that harboured a single Vice sequence variant each. It is notable that the Vice variants were always identical in strains of the same MST sequence type. To verify whether the non-synonymous mutations in the gene sequence of Vice were associated with additional silent mutations, the SNPs found in the gene locus of Vice were checked. Remarkably, the vast majority of mutations in the gene were missense variants. Synonymous base exchanges were only found in four strains: CbuG_Q212 (GGV), CB149 (GGIV-a), CB202 (GGIV-a) and Cb3506 (GG_NA).

### T4BSS protein sequence variation

Similar to the effector proteins, there was considerable variability in the protein sequences of the T4BSS in the strains investigated (Supplementary Fig. 4, Supplementary Table 8). These protein variants largely coincided with the GG. Again, the intra-GG variability was highest in GGIV and GGV. IcmH was disrupted in GGIII. IcmV was truncated at the C-terminal end in all GGs except for GGII-a. Only IcmT and IcmR were conserved across all GGs. Also, DotN and IcmL2 were highly conserved, as protein sequence variations were only observed in three strains each (CbuG_Q212, DOG UTAD, Scurry_Q217 and 22QC1336, CbCVIC1, CB13, respectively).

## Discussion

*Coxiella burnetii* exhibits a broad host range, and the outcome of infection can vary considerably [2, 41, 42]. While the bacterium infects both small and large ruminants, human Q fever outbreaks are almost exclusively linked to shedding by sheep and goats. Human infections are rarely linked to cattle, even though the seroprevalence is high in cattle [43]. The differences in disease manifestation led to the hypothesis of isolate- and, later, GG-specific virulence or host adaptation [4, 5, 9, 10]. However, several studies contradict this hypothesis and indicate that host factors contribute to the disease outcome [44–46]. The ability of *C. burnetii* to invade and persist in the host cell is, besides the expression of a full-length smooth lipopolysaccharide, facilitated by the production and secretion of effector proteins which interfere with or modulate host cell processes [47, 48]. In the present study, we aimed at characterizing *C. burnetii* strains based on genomic features and differences in their effector protein repertoires. By adding genome data from strains of animal origin to the publicly available, human-dominated dataset, we applied a One Health approach to coxiellosis and the potential role of *C. burnetii* effector proteins in host specificity.

Our analyses showed that several genomic traits of *C. burnetii* were consistent with the classification of the agent into GGs. These Groups were also congruent with sequence types determined by MST, as shown by the results presented here and by others [6, 7]. However, not all of the known GGs were present in the dataset investigated, i.e. GGI-b and GGII-d as well as GGVII and GGVIII were missing due to the lack of good-quality sequencing data.

GGs are assumed to be associated with a preferred host species and specific disease manifestations in humans, as GGI-III were predominantly found in patients with acute Q fever, whereas GGIV and GGV were mostly connected to chronic cases [4]. In agreement with previous findings [5, 6], the results presented showed that GGIII was dominated by cattle isolates, while most goat and sheep isolates belonged to GGII-a and GGII-b. The groups GGIV-a, GGIV-b and GGV were human isolate-dominated and associated with chronic Q fever as described before. The difficulty in correlating disease phenotype with isolate sequence identity is nicely demonstrated by GGII-c which was originally associated with acute human Q fever cases, but was dominated in this study by isolates from human chronic Q fever cases. A recent study from Spain has shown that similar *C. burnetii* genotypes can lead to acute as well as chronic disease outcomes in humans [49], calling into question the link between GG and Q fever manifestation. Interestingly, MST ST8 (GGIV-a) had been linked to goats before, as it was detected in caprine milk in the USA [50], but only two goat isolates from the dataset investigated here fell in this cluster. Considering the fact that coxiellosis is primarily an animal disease, the over-representation of good-quality sequencing data from human chronic Q fever specimens in the publicly available databases can be considered a sampling bias. Despite *C. burnetii* being endemic almost worldwide, there is a lack of comprehensive sequence data, particularly from animals. By adding 36 samples of animal origin from Germany, we aimed to reduce this bias. The results displayed here show that most genomic groups are found in multiple host species, albeit with some level of dominance for certain species. This host range may become even more diverse with increasing availability of sequence data from isolates of animal and human origin, geographical source or different disease outcome. Multiple hosts in each GG suggest a certain degree of host flexibility and adaptability of *C. burnetii* rather than strict host specificity.

The core genome comprises all genes that are present in all genomes of a species, but its composition can vary considerably depending on the strains included in the analysis. It has to be noted, that a gene's absence in an assembly could also result from assembly errors and might not reflect true biological loss. Here, we found 2237 genes in 140 *C. burnetii* genomes, 1624 of which constituted the core genome genes present in at least 99% of the strains, i.e. 139 isolates. In previous studies, the core genome of *C. burnetii* was estimated to be smaller. Hemsley et al. [5] found 1311 or 989 core genome genes among 67 isolates, depending on the bioinformatic pipeline applied, whereas Abou Abdallah et al. [12] found a considerably higher number of total genes (*n* = 4501) and a similar number of genes in the core genome (*n* = 1211) among 75 *C. burnetii* genomes. It has to be noted that several genomes that were included in the dataset of the latter study were excluded here as they failed quality control. This highlights the impact and importance of rigorous quality screening of genomic data prior to analysis. Besides the quality of the input data, the number of core genes also varies with the methodological choice, due to pangenome tool-dependent biases [51, 52]. There are various approaches to pangenome estimation [53], e.g. based on nucleotide and/or amino acid sequences, that influence the number of detected core genes. However, Panaroo, that was used here, proved to give consistent and robust results for *Mycobacterium tuberculosis* [51], another bacterial agent with a high level of genome conservation.

In contrast to other GGs, the GGVI (Dugway strains) were found to be attenuated or avirulent in most hosts [54]. In agreement with other reports, these strains had the largest genomes of all groups. However, in contrast to a previous study [32], they did not have the lowest pseudogene content. Based on the Bakta annotation, the number of pseudogenes in NMI was almost half of that reported in the initial publication of the complete NMI genome [11], which identified 83 pseudogenes. The Dugway isolates, however, had fewer hypothetical genes, i.e. genes of unknown function, than NMI. Genome degradation usually leads to the formation of pseudogenes, but annotation pipelines might also classify them as hypotheticals, which could explain the discrepancy. The comparability of genome studies can be hampered by the usage of different annotation tools and concomitant differences in CDS annotation. As demonstrated for NMI, known sequences, such as effector-coding genes, might not be found in new annotations or open reading frames can differ in their extent.

The larger genome size and higher core gene content observed here, together with the observed avirulence in most hosts, could explain why GGVI is considered closer to the last common ancestor in the *C. burnetii* phylogeny than the other GGs [32, 55]. These other lineages might have emerged as consequence of the pathogen’s introduction into other hosts, resulting in gene decay. Genome reduction is a common phenomenon during the evolution of obligate intracellular bacteria as the selective pressure on genes, that are not required, is reduced, ultimately leading to their loss. It is hypothesized that this process is still ongoing in *C. burnetii* [11] and it might even increase the pathogen’s virulence [56]. When there is a shift in a pathogen’s virulence towards a host, usually similar levels of susceptibility and virulence can be expected in phylogenetically closely related hosts, as shown for viruses [57]. This agrees with the apparent host preferences of GGs, i.e. ruminant- and human-dominated lineages. However, other hosts can also be susceptible, as the host’s health state influences the disease outcome, which could account for human infections by lineages that are dominantly found in ruminants.

Among the dataset investigated, the difference of strain Namibia to other strains of the same GG, GGIV-b, was striking. Based on the findings presented, it can be assumed that MST ST30, to which strain Namibia belongs, could represent another sub-group of GGIV, as it has a characteristic set of SNPs and effector protein variants.

Compared to other intracellular bacteria, *C. burnetii* has a high IS element content with considerable variation between individual strains within the same Genomic Group. Some elements, like ISNCY (“IS not classified yet”) and IS1111 are conserved across all lineages, even with respect to their insertion site [58], while others were only rarely detected, like IS4, which is known to be located on the QpDG plasmid of the Dugway strains [32], and IS481. Surprisingly, IS1111, which is commonly used as target for *C. burnetii* diagnostic PCR [59], was not detected in three strains. This could be a false-negative result caused by sequence deviations from the reference sequence of the IS1111 elements in these strains, as deletions in IS1111 copies have been noted before [58]. However, some authors also reported *C. burnetii* strains lacking IS1111, which originated from marine mammals, e.g. in Australia and Alaska [60, 61]. Here, the strains that lacked IS1111 came from a dog from Canada and human patients from Switzerland and Romania, respectively. IS1111 is particularly interesting because Seshadri et al. [11] found a genomic locus resembling a pathogenicity island connected to IS1111 elements, indicating that they might be involved in pathogenicity. As the insertion and excision of insertion elements can modulate gene expression [62], combined with our finding, that the IS content varies considerably even within GGs, indicates that the role of IS elements in *C. burnetii* virulence warrants further in-depth investigation.

Since the effector protein repertoire might impact disease manifestation and host preference, the presence and absence of known effector proteins in the 140 *C. burnetii* strains was investigated. In agreement with previous studies [18], the strains of GGVI harbored several unique effectors. According to a study by Metters et al. [63], who used transposon library screening for determining essential genes in *C. burnetii*, there are 512 genes essential for survival of the reference strain Nine Mile I in axenic medium, among which there were also 12 effector-coding genes. These 12 essential effectors were also detected in the current study, although not always in every GG. Here, we identified five effector proteins with highly conserved sequences among all strains: Cem8 (CBU_1634a), CBU_0469, CBU_1314a (not in GGV), CBU_1594 (MceD), and CBU_2076. None of these were identified as essential by Hemsley et al. [63]. However, genes essential for survival in vivo might not always be necessary for survival in axenic medium. The high level of conservation of the five effector proteins suggests important roles for these proteins in *C. burnetii* virulence. However, experimental evidence is lacking.

Additionally, five effector proteins were investigated in detail, CBU_0077 (MceA), CBU_0513 (CinF), CBU_0781 (AnkG), CBU_0822 (CbFic2) and CBU_2007 (Vice). These have been proven to be translocated into the host cell in a T4BSS-dependent manner and host cell targets or pathways have been identified, except for CBU_0822. They interfere with apoptosis and host cell transcription, and/or are essential for intracellular replication and CCV biogenesis. All the effector proteins analyzed in depth displayed different degrees of sequence variation, with varying severity of effect. For example, the C-terminal region of MceA was conserved among all isolates analyzed ensuring translocation into the host cell [64]. However, several amino acid substitutions were found within the N-terminal region which may affect its function. Contrary, CinF, AnkG and CbFic2 displayed low to moderate numbers of sequence variants, but several of these variants featured deletions in their C-terminal or N-terminal region or a frame shift, that could impede translocation or function. Interestingly, most sequence variations in MceA, CinF, AnkG and CbFic2 were found in isolates from GGIV and/or GGV, which are associated with chronic Q fever. The effector protein Vice showed a high level of sequence variation across most Genomic Groups. Strikingly, most missense variations were found in GGI, GGII and GGIII isolates and synonymous mutations in GGIV and GGV isolates. This might indicate an ongoing adaptation process in isolates of GGIV and GGV due to a chronic or persistent phase of infection. Therefore, a persistent, low activity phase in the host for long periods of time may create selection pressure promoting genetic adaptation and diversification e.g. of the here analyzed T4BSS effector proteins [65, 66]. However, this assumption may be biased due to the limited availability of data from acute Q fever cases and cannot be proven or disproven on the basis of our data. It should be noted that the repertoire of effector proteins investigated in this study only included experimentally confirmed effectors, as determined by a literature search. By applying specific tools for in silico effector protein detection, such as S4TE [67] and T4SEpp [68], it can be assumed that several more putative effector proteins can be identified. Further investigation and experimental validation is needed to establish whether these represent true effectors and if they contribute to host specificity.

A functional effector protein secretion system is a prerequisite for host infection, i.e. pathogenicity. Thus, we expected the T4BSS components to be highly conserved. To our surprise, these proteins also displayed a considerable level of variability, particularly in GGVI-a and GGIV-b. Not all 24 T4BSS proteins have known functions. Some might be dispensable, e.g. IcmH, which was disrupted in GGIII. For the T4BSS proteins, the localization of mutations must be investigated in detail, as some mutations might not affect functionally important regions and therefore should not interfere with the protein’s function. 

In vivo studies showed that the pathogenicity and virulence of *C. burnetii* correlate with genomic lineages [9, 10, 69], and that GGs harbor specific gene inventories and nucleotide polymorphisms, e.g. deletions [5, 8]. The results presented here were all in agreement with the hypothesis that effector protein variants are connected to genomic lineages, rather than hosts. From our analyses neither the mere presence or absence of effector genes nor the occurrence of specific SNPs can be correlated with the host species. However, only the core genome region of the strains was considered in the SNP analysis. Thus, SNPs in genes, that were absent from NMI or other genomes included in the dataset, were not analyzed. Comprehensive investigations are complicated due to the lack of sufficient genomic data as well as metadata, i.e. a human infection could be attributed to contact with an animal source or if animals were held in mixed herds. The latter was previously demonstrated, where a cattle-associated genotype caused abortion in goats [70]. The lack of metadata is a notable limitation in this study. If a well-defined dataset was available, other factors, like virulence, pathogenicity or tissue tropism could be included in the analysis as potential factors for host preferences.

As our analysis results suggest that no single effector determines host specificity, what else could determine host preference or disease manifestation? One possibility is that transcriptional regulation of effector protein-coding genes and potential effector synergetic effects might play a role. Furthermore, there could be yet undiscovered effector proteins and virulence determinants. Besides, several other factors have been hypothesized to influence disease outcome, such as plasmid presence and LPS chemotype [4, 12, 71]. Disease manifestation additionally depends on the individual immune status of the host. Predisposed patients with an existing heart condition, immune suppression or pregnant women are more likely to develop chronic Q fever [44, 45]. The release of cytokines, such as tumor necrosis factor-alpha (TNF-α) and interleukin (IL)-10, has been linked to Q fever endocarditis, whereas the release of TNF-α, interferon-gamma (IFNγ) and IL-6 was observed in human acute Q fever [72–74].

However, infections in ruminants have a broad spectrum of clinical outcomes, with abortion being more frequent in goats than in sheep and rare in cattle [75]. Placentation type (synepitheliochorial) of cattle, sheep and goats are very similar, with trophoblasts migrating and fusing with maternal epithelial cells (syncytium) building a stable interface [76, 77]. In these host species, *C. burnetii* exhibits a tropism for the reproductive organs and replicates within the trophoblast layer [78, 79]. This cell type is essential for immune suppression and tolerance by secretion of steroids and hormones to avoid embryonic loss. It was shown that progesterone has an inhibitory effect on *C. burnetii* replication in human trophoblasts (JEG-3) [80]. Thus, hormone levels or the individual immune status of the host may influence pregnancy outcome.

## Conclusions

This study highlights the genomic diversity of *C. burnetii*, between and within distinct GGs which could imply preferences for certain hosts. Effector protein profiles were found to correspond to genomic lineage rather than to host origin. In-depth analyses of selected effectors (e.g. AnkG, CbFic2, CinF, MceA and Vice) demonstrated that specific amino acid substitutions and truncations may influence protein localization and activity, potentially affecting virulence. However, no single effector gene or mutation could be definitively linked to host specificity. The data emphasize the need for broader, high-quality genomic and functional datasets, particularly from animal sources and human acute Q fever cases, to resolve the multifactorial determinants of host adaptation and pathogenesis in *C. burnetii*. Transcriptional regulation, effector interactions, and additional virulence factors, such as plasmid type and LPS chemotype, along with host immune responses, likely play critical roles in the adaptability of *C. burnetii* to host species, and contribute to disease outcome.

## Methods

### Data acquisition and quality control

The Short Read Archive of NCBI was browsed (accession date: 12.07.2024) for *C. burnetii* data with the criteria „DNA“, „Illumina“ and „paired“. The resulting data was downloaded and the quality was checked using the WGSBAC pipeline v2 (https://gitlab.com/FLI_Bioinfo/WGSBAC/-/tree/version2) by determining the coverage, assembling the genomes using Shovill, assessing assembly quality with QUAST, and checking reads and assemblies for contamination using Kraken2. The following thresholds were used as exclusion criteria: coverage < 30X, assembly size > 2.2 Mb, total number of contigs > 120, GC% >42.8%, GC% <42.3%, Kraken2 best match for reads not *Coxiella* or less than 90% *Coxiella* and Kraken2 best match for contigs < 90% *Coxiella*. Contamination with human DNA was considered tolerable, if it was not dominant (> 50%).

Further, RefSeq was browsed for *C. burnetii* genomes and the assemblies downloaded for quality control. The assembly statistics were assessed using QUAST v5.2.0 [81]. Kraken2 v2.0.7_beta [82], CheckM v1.2.3 [83] and BUSCO v5.7.1 [84] were used for inter- and intraspecific contamination detection, respectively, as well as for a completeness check. Genomes that showed less than 85% complete BUSCOs of the legionellales_odb10 were excluded.

### Strain cultivation and DNA isolation

To complement the publicly available dataset, *C. burnetii* strains from the National Reference Laboratory for Q Fever, Germany, of the Friedrich-Loeffler-Institut were chosen. These were cultivated under biosafety level 3 conditions in ACCM-2 as previously described [85, 86]. Briefly, 500 ml of ACCM-2 were inoculated with 1e + 05 bacteria/ml and incubated for 7 days at 37 °C with 5% CO_2_ and 2.5% O_2_. Bacteria were harvested by centrifugation at 15,000 x g and 4 °C for 20 min. Bacterial pellets were resuspended in 1 ml sucrose glycerol buffer (270 mM sucrose, 10% glycerol) and stored at -80 °C. DNA was extracted from 20 µl bacterial suspension using the QIAamp DNA mini Kit (QIAGEN GmbH, Hilden, Germany) as recommended by the manufacturer. Bacteria were quantified by real time PCR (qPCR) using the isocitrate dehydrogenase encoding gene (*icd*) as target as previously described [87].

### Whole genome sequencing and genome assembly

Bacterial biomass was resuspended and inactivated in DNA/RNA Shield buffer (Zymo Research Europe GmbH, Freiburg, Germany) and sent for DNA extraction and subsequent whole genome sequencing to MicrobesNG (Birmingham, United Kingdom). Short-read libraries were prepared with the NexteraXT kit (Illumina Inc., San Diego, USA) and sequencing was conducted on a NovaSeq6000 machine. Adapters were subsequently trimmed from the reads using Trimmomatic v0.30 [88] with a sliding window quality cutoff of Q15. Additionally, long-read Nanopore sequencing was conducted using the Rapid Barcoding Kit (SQK-RBK114.96) (Oxford Nanopore Technologies Ltd, Oxford, United Kingdom) for library preparation. The libraries were loaded on an R10.4.1 type flowcell (FLO-MIN114) and sequenced on a GridION device. Basecalling was done directly on the GridION using the high-accuracy model dna_r10.4.1_e8.2_400bps_hac@v4.3.0.

The long- and short-read data was assembled using the BONT pipeline (as of 25.7.2024) (https://gitlab.com/FLI_Bioinfo/BONT). As assemblers, Flye v2.9.4-b1799 [89] with the --meta option to account for coverage differences between plasmid and chromosome and Unicycler v0.5.0 [90] were chosen to cover a long-read- and a short-read-first approach. In both approaches, the assemblies were polished using Illumina reads by polyPolish v0.6.0 [91]. The quality of the assemblies was checked as described above. The assembly approach using the flye assembler yielded complete genomes comprising the chromosome and a plasmid. There was only one exception, *C. burnetii* strain 18QC1770, for which the coverage of the long reads was not sufficient for the long-read first approach, which is why Unicycler was used for assembly, yielding a genome with 36 contigs.

The raw sequencing data and assemblies were deposited with the European Nucleotide Archive under the project number PRJEB88958.

### Multispacer sequence typing and single nucleotide polymorphism analysis

For in silico multispacer sequence typing (MST), a database with the spacer sequences was built using ABRicate v1.0.1 (https://github.com/tseemann/abricate) from the alleles available at https://ifr48.timone.univ-mrs.fr/mst/Coxiella_burnetii/spacers.html (accessed on 02.08.2024). The assemblies were screened and the resulting profiles were browsed in CoxBase (https://coxbase.q-gaps.de/webapp/) [92] for assignment of a sequence type (ST).

Single nucleotide polymorphisms in the core genome region (cgSNPs) were determined using Snippy v4.6.0 (https://github.com/tseemann/snippy). Where available, raw sequencing data was used. For genomes, that were only available as assemblies, the –contig option was used. The *C. burnetii* strain Nine Mile I (GCF_000007765.2) served as reference genome. The output was an alignment of all core genome SNP positions, which was analysed by maximum likelihood analysis using RAxML v8.2.12 [93] (raxmlHPC-PTHREADS -m ASC_GTRCAT --asc-corr=lewis -V -N autoMRE -p 12345 -x 12345 -f a). The SNP differences between strains in this alignment were counted by the script snp-dists v0.8.2 (https://github.com/tseemann/snp-dists) that created a distance matrix, which was used for cluster analysis with the hclust function in R [94]. Maximum likelihood trees from different approaches were compared using the tanglegram function of Dendroscope v3.5.9 [95]. The effects of SNPs at the amino acid level were checked using snpeff as implemented in Snippy.

### Genome characteristics and pangenome analyses

The assemblies were annotated using Bakta v1.9.3 with database v5.1(full) [96] and a pangenome analysis was conducted using Panaroo v1.4.2 [52]. The resulting filtered core genome alignment was used for maximum likelihood analysis and the construction of a phylogenetic tree by RAxML v.8.2.12 (parameters: -m GTRGAMMA -p 2352890 -# 100). The tree and the pangenome presence/absence matrix were visualized by Phandango [97]. Further, a Neighbour Joining analysis was done based on the presence and absence of accessory genes with GrapeTree v1.5.0 [98].

Insertion sequences (IS elements) were detected using ISEScan v1.7.2.3 [99]. Boxplots were created in Python using the Seaborn package v0.13.2 [100].

### Effector protein and T4BSS screening and comparison

The positions of the coding sequences of the NMI effector proteins identified in the literature search were extracted from the RefSeq annotation files (Additional file 7 - Figure S2). The start and stop positions of these coding sequences were compared to the new Bakta-based ORF positions of GCF_000007765.2. Most of the effector ORFs (*n* = 118) of this new Bakta-based annotation of NMI were identical to the original RefSeq annotation. However, for 12 effectors, the newly annotated ORFs started or ended at a different position and for one effector (CBU_0375, formerly annotated as pseudogene) no new counterpart was identified, as neither start nor end position was identical to the original annotation.

Besides the protein sequences, the gene sequences of the NMI effector proteins were also extracted from the RefSeq annotation file. The sequences were searched for in four reference strains (GCF_000017105.1, GCF_000019865.1, GCF_000019885.1, GCF_000018745.1) using the BLASTn online service [101]. Homologous genes were listed (Additional file 2 - Table S2) and the corresponding protein sequences downloaded from NCBI RefSeq.

For screening the annotated genomes in the dataset studied, a database of all effector proteins found in the reference genomes was created that was used for screening with Diamond v2.1.8 [102] (parameters: query coverage 80%; subject coverage 40%; sequence identity 80%). The translation products of the CDSs, that were identified as potentially effector protein-coding, were extracted from the Bakta annotation files using seqkit v2.9.0 [103] and separate alignments were created for each effector by MAFFT v7.520 [104] using the –auto option. The sequences of each alignment were assigned to clusters for determining identical sequence types using the SciPy Python package v1.14.1 (scipy.cluster.hierarchy function), as well as potential truncations [105]. The alignments were visually checked to confirm the truncation classification.

The result of this analysis was visualized in Microreact [106] together with the phylogenetic tree generated in the pangenome analysis. Further, the data was subjected to Neighbour Joining analysis as described before.

The components of the T4BSS were compared in a similar manner. For this, the genes encoding the T4BSS components (*n* = 24) were taken from the virulence factor database [107]. Corresponding protein sequences were extracted from the RefSeq annotation and used for creating a database, which was used for screening the Bakta annotation of NMI. Then, the corresponding lines were extracted from the pangenome gene presence/absence table and all corresponding protein sequences from all strains investigated were compared as described above.

## Supplementary Information

Additional file 1: Table S1. Strains used. List of strains used in this study with corresponding metadata and results of genome data quality analysis as well as MST analysis.

Additional file 2: Table S2. Known *C. burnetii* effector protein-coding genes. List of accession numbers of known effector protein-encoding loci in *C. burnetii* strain Nine Mile I. Corresponding loci in other reference strains were identified by BLAST search.

Additional file 3: Figure S1. Boxplots Chromosome size. Box plot showing the distribution of genome assembly sizes of all investigated *C. burnetii* genome assemblies (*n* = 140). Shapes and filling of the data points indicate the host of the strain.

Additional file 4: Table S3. Detected IS elements. Typed and corresponding numbers of IS elements detected in 71 *C. burnetii* genome assemblies that were almost closed (max. three contigs).

Additional file 5: Table S4. Number of core genes per Genomic Group. Total number of genes shared by all strains of each Genomic Group and relative percentage com-pared to the mean of detected genes in all strains of a Genomic Group. Groups II and IV have been split into subgroups and are only mentioned for backward comparison.

Additional file 6: Table S5. Unique SNPs in core genome and effector genes. Location of unique base variants per Genomic Group detected in either the complete core genome or the effector genes that were present in all strains. The positions are relative to the reference strain Nine Mile I (GCF_000007765.2).

Additional file 7: Figure S2. Screening for effector proteins. Workflow used in the study for detecting homologous genes and proteins of the effector-coding genes listed in Additional file 2 - Table S2. The figure was created with BioRender (https://biorender.com/).

Additional file 8: Table S6. Sequence variations of detected effector proteins. Sequence variants of T4BSS effector proteins detected in the investigated *C. burnetii* strains. A num-ber was assigned to each variation.

Additional file 9: Figure S3. NJ tree effector proteins. Neighbor joining tree based on the results of sequence variations of effector protein sequences detected in the investigated strains (see Additional file 8 - Table S6).

Additional file 10: Table S7. Unique effector protein variant per Genomic Group. Unique sequence variants of effector proteins per Genomic Group. The designation of the proteins is according to the proteins’ similarity to reference strain Nine Mile I (GCF_000007765.2) effectors.

Additional file 11: Figure S4. T4BSS protein variants. Presence and status of T4BSS proteins in 140 *C. burnetii* strains. Block colors indicate differences in amino acid sequence, i.e. blocks with identical colors for one protein show sequence identity. Host species, Genomic Group affiliation and truncation and disruption are indicated as given on the right side of the figure.

Additional file 12: Table S8. T4BSS protein sequence variants. Sequence variants of T4BSS proteins detected in the investigated *C. burnetii* strains. A number was assigned to each sequence variation.

## Data Availability

The datasets generated during the current study are available in the European Nucleotide Archive un-der project number PRJEB88958.

## Abbreviations

PCR: Polymerase chain reaction
GG: Genomic Group
MST: Multispacer sequence typing
ST: Sequence type
NMI: Strain Nine Mile I
cgSNP: Core genome single nucleotide polymorphism
SNP: Single nucleotide polymorphism
ORF: Open reading frame
DNA: Deoxyribonucleic acid
T4BSS: Type IVB secretion system
CCV: Coxiella–containing vacuole
