# Supplementary figures and images for: Genome characteristics and type IV effector protein repertoire of Coxiella burnetii depend rather on Genomic Groups than on host species
Source: BMC Microbiol. 2026 Apr 22;26:393. doi: 10.1186/s12866-026-04897-w (PMC13104383; doi:10.1186/s12866-026-04897-w)

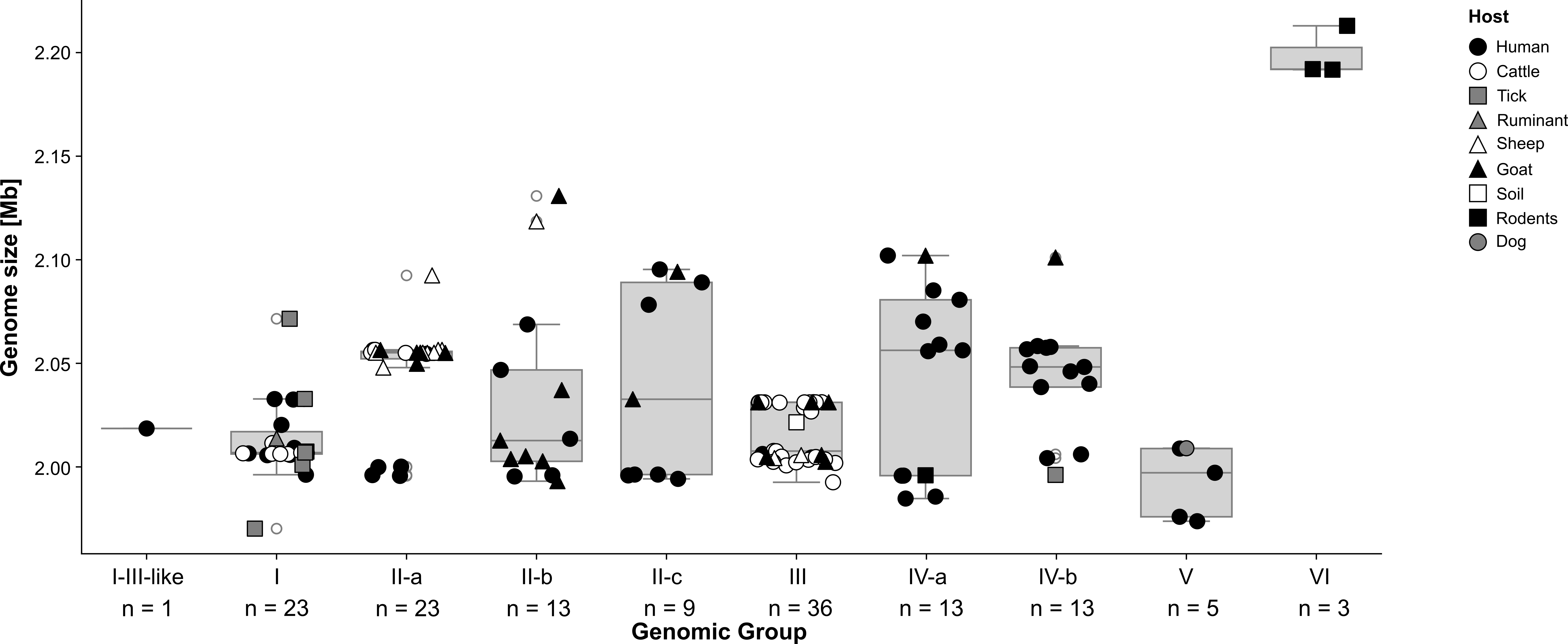

Supplement: Supplementary file 3 — Additional file 3: Figure S1. Boxplots Chromosome size. Box plot showing the distribution of genome assembly sizes of all investigated C. burnetii genome assemblies (n = 140). Shapes and filling of the data points indicate the host of the strain. [file 12866_2026_4897_MOESM3_ESM.png]

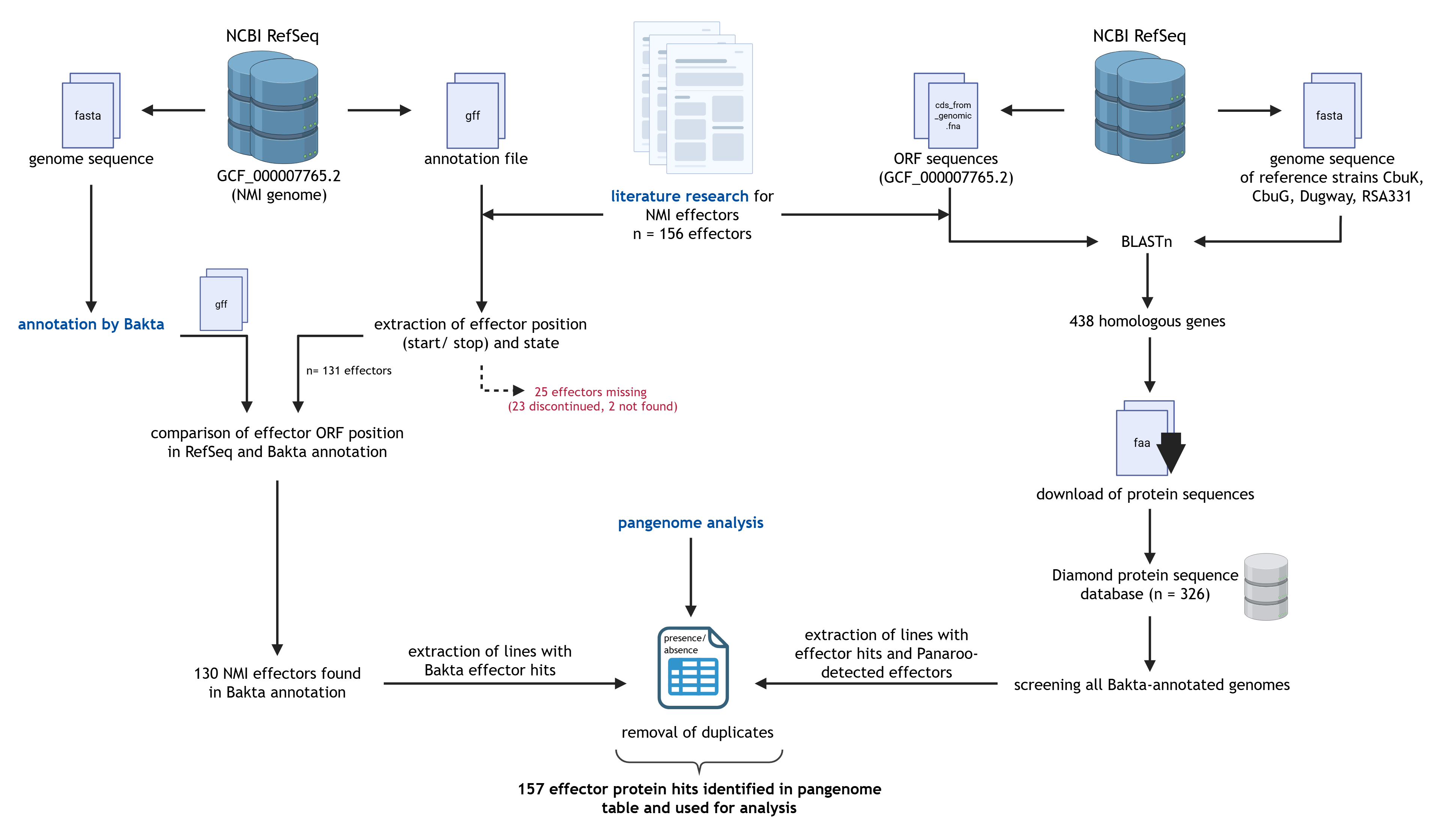

Supplement: Supplementary file 7 — Additional file 7: Figure S2. Screening for effector proteins. Workflow used in the study for detecting homologous genes and proteins of the effector-coding genes listed in Additional file 2 - Table S2. The figure was created with BioRender (https://biorender.com/). [file 12866_2026_4897_MOESM7_ESM.png]

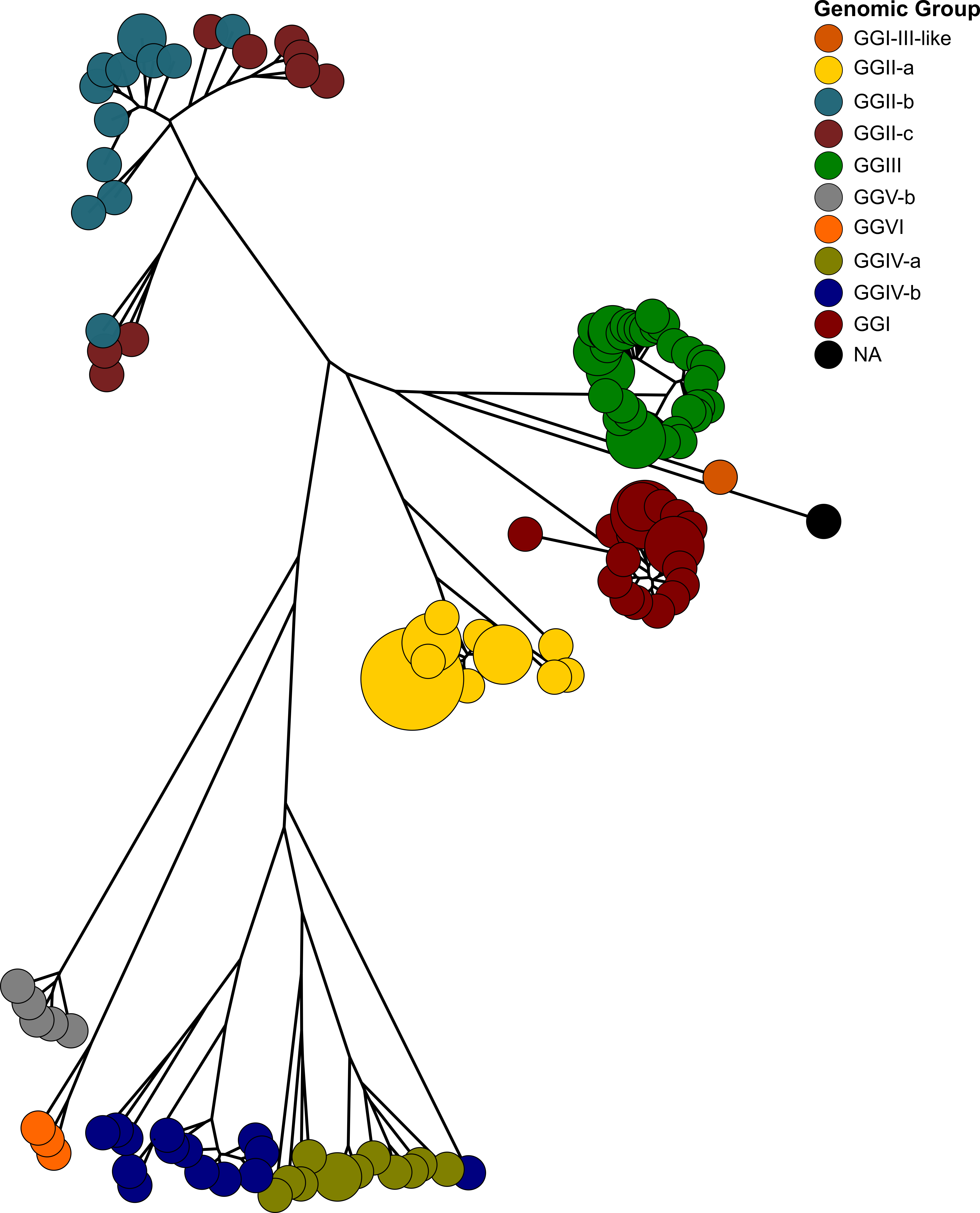

Supplement: Supplementary file 9 — Additional file 9: Figure S3. NJ tree effector proteins. Neighbor joining tree based on the results of sequence variations of effector protein sequences detected in the investigated strains (see Additional file 8 - Table S6). [file 12866_2026_4897_MOESM9_ESM.png]

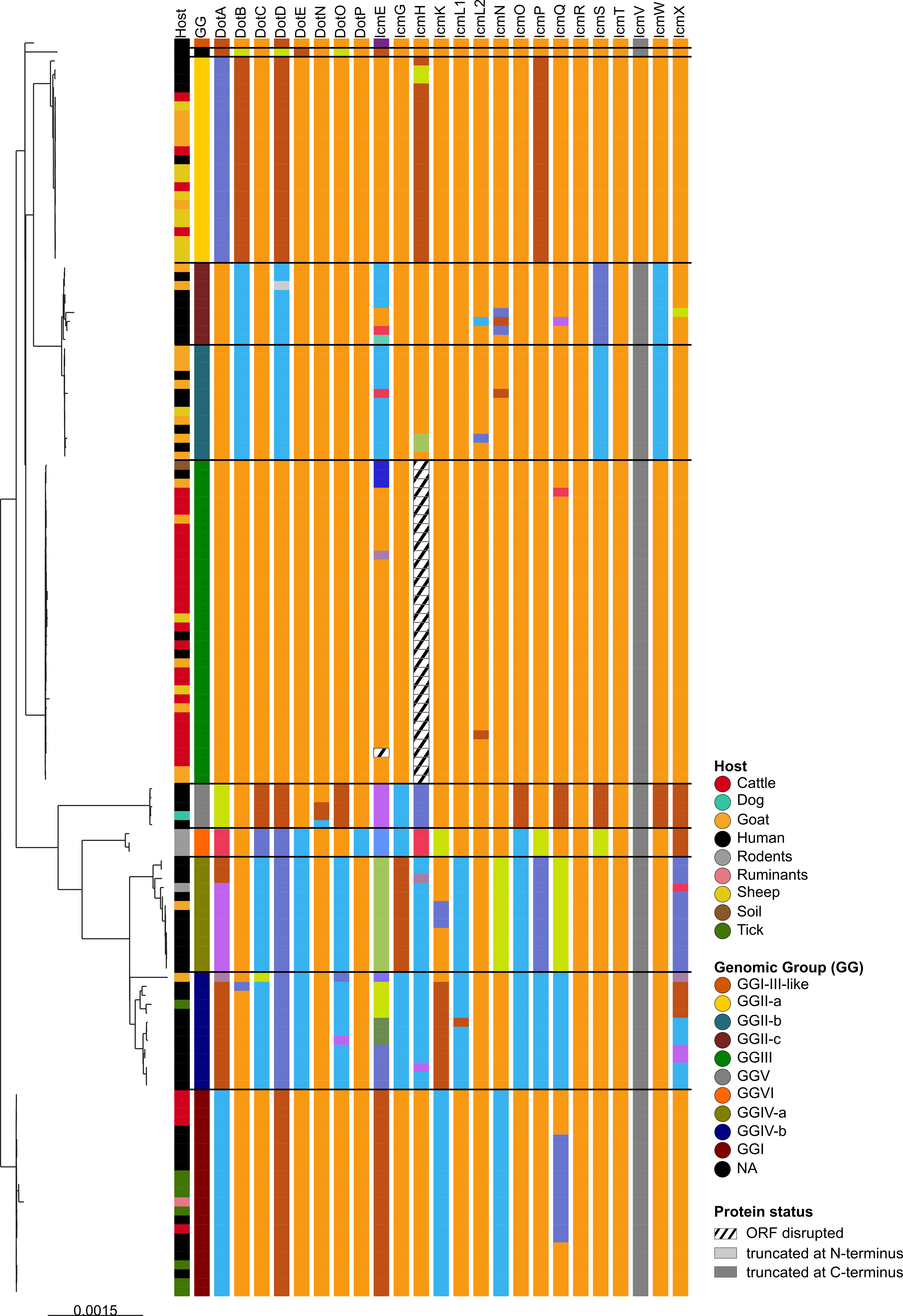

Supplement: Supplementary file 11 — Additional file 11: Figure S4. T4BSS protein variants. Presence and status of T4BSS proteins in 140 C. burnetii strains. Block colors indicate differences in amino acid sequence, i.e. blocks with identical colors for one protein show sequence identity. Host species, Genomic Group affiliation and truncation and disruption are indicated as given on the right side of the figure. [file 12866_2026_4897_MOESM11_ESM.png]
